# Serology Confirms Near-Absence of Malaria Transmission in Timor-Leste

**DOI:** 10.4269/ajtmh.25-0700

**Published:** 2026-08-27

**Authors:** Elfiana De Fatima Amaral, Isabel Byrne, Helena Brazal Monzo, Raul Sarmento, Maria Do Rosario De Fatima Mota, Gregorio Rangel, Maria Tanesi, Antonio Salles de Sousa, Nelson Martins, Tessa Oakley, Lucsendar Alves, Jennifer Yan, Paul Arkell, Joshua R. Francis, Chris Drakeley

**Affiliations:** ^1^ Menzies School of Health Research, Charles Darwin University, Dili, Timor-Leste;; ^2^ Associação Maluk Timor, Dili, Timor-Leste;; ^3^ Department of Infection Biology, London School of Hygiene and Tropical Medicine, London, United Kingdom;; ^4^ Communicable Disease Control Department, Ministry of Health, National Directorate of Public Health, Dili, Timor-Leste;; ^5^ Ossomali Research and Development, Dili, Timor-Leste

## Abstract

Despite malaria remaining a major public health threat, several countries have made significant progress toward elimination. Timor-Leste has reduced the number of malaria cases from more than 200,000 to zero, with no indigenous cases reported since 2022, and has recently been certified as malaria-free by the WHO. At low transmission levels, serological surveys, which detect antibodies to malaria parasites, can augment conventional surveillance by revealing the absence of antibodies and, therefore, of exposure in previously endemic populations. Exposure to malaria, as determined using antibody responses to antigens from *Plasmodium falciparum* (*P. falciparum*) and *Plasmodium vivax* (*P. vivax*), was assessed in 4,631 participants recruited from all municipalities in Timor-Leste. These antigens are associated with recent and cumulative exposure to each species. Age-specific seroprevalence was assessed, and seroconversion was estimated using reverse catalytic models. Risk analysis for individual and geographical exposure was also conducted. Seroprevalence to recent exposure to *P. falciparum* antigens was uniformly low across age groups (1.4% [95% CI: 1%–2%]) and consistent with the absence of ongoing transmission. The recent seroprevalence of *P. vivax* was similarly low (0.2% [95% CI: 0.1%–0.3%]). The time of change in the force of infection was consistent with the country’s scale-up for elimination. Areas at lower altitudes and those with higher historical tree cover had evidence of the highest co-endemic historical exposure. The serological analysis supports passive and active surveillance malaria data in confirming the near-absence of ongoing transmission in Timor-Leste. In low-transmission settings, sero-epidemiological surveillance can provide key information on transmission that will benefit control programs.

## INTRODUCTION

Malaria remains a major public health threat in many parts of the world, primarily affecting young children and pregnant women in sub-Saharan Africa.^1^ However, several countries have made significant progress toward elimination, with the notable addition of China in 2021. The WHO grants certification of malaria elimination when there has been a nationwide interruption of local human malaria transmission for at least 3 consecutive years, along with a fully functioning surveillance and response system to prevent the reestablishment of indigenous transmission.^2^

Timor-Leste, a country in Southeast Asia, occupies the eastern part of the island of Timor, the largest island of the eastern Lesser Sunda Islands in the Malayan Archipelago.^3^ The country is 15,007 square kilometers in size, with a central mountainous area surrounded by coastal plains. A census in 2022 estimated the population to be 1.34 million.^4^ Malaria was ranked second on the list of the top 10 priority diseases almost two decades ago, with more than 200,000 cases being reported in 2006 and 2007.^5^ Both *Plasmodium falciparum* (*P. falciparum*) and *Plasmodium vivax* (*P. vivax*) were prevalent in Timor-Leste, with the former accounting for more than 70% of confirmed cases.^5^ *Anopheles barbirostris* (*An. barbirostris*) and *Anopheles subpictus* (*An. subpictus*) were identified as primary and secondary vectors.^6^ Both *An. barbirostris* and *An. subpictus* were associated with coastal areas in West Timor.^7^

The Timor-Leste National Malaria Program (NMP) was implemented nationwide in 2006 and underpinned by improved case detection and vector control strategies. The program resulted in a 97% decrease in the reported malaria incidence: from 223,002 cases in 2006 to 6,202 cases in 2012.^5^ Since 2016, the vast majority of cases have been imported from Indonesia,^6^ and the last indigenous cases were reported in 2020 in the bordering regions of Oecusse Municipality.^8^ The last recorded malaria deaths were in 2015.^1^ Timor-Leste reported no indigenous malaria cases in 2022, and the country was granted malaria-free status in 2025.^9^

Serological surveillance, the measurement of antibodies against specific pathogen antigens, is widely used to estimate population levels of exposure to infection. In malaria, the identification of antigens associated with recent and historical exposure allows for a more nuanced examination of risk for and patterns of exposure.^10^^,^^11^ The aim for the present study was to make use of the nationwide survey (Vaccine Preventable Disease Seroprevalence in a Nationwide Assessment of Timor-Leste [VASINA-TL])^12^ conducted between 2021 and 2022 to examine population-level exposure *to P. falciparum and P. vivax* in the context of the country’s malaria elimination efforts.

## MATERIALS AND METHODS

### Survey.

Samples were collected in a population-representative, national cross-sectional serological survey conducted in all 13 municipalities of Timor-Leste as part of VASINA-TL. The survey protocol is described in Arkell et al.^12^ The survey was conducted between September 2021 and October 2022. Briefly, a three-stage cluster random sampling strategy was used. Firstly, a prespecified number of enumeration areas (EAs) per municipality was selected, with probability proportional to each municipality’s population. A prespecified number of households within each EA were selected. Finally, all occupants in the selected households who met the eligibility criteria were invited to participate. Each participant completed a structured questionnaire covering basic demographic, clinical, and vaccine-related information. A venous blood sample was collected into a serum separator tube, centrifuged to obtain serum, and refrigerated in the field.

### Serological analyses.

Samples were tested using a multiplex bead-based immunoassay as described previously.^13^ Briefly, specific MagPlex microsphere bead regions (Luminex Corp. [Austin, TX, USA]) were chemically coupled to previously optimized antigen concentrations. The antigen panel included a total of 12 malaria antigens. For *P. falciparum*, Etramp5Ag1, Gexp18, HSP40, and HYP2 were used to define recent exposure, and GLURPRII, *Pf*AMA1, and *Pf*MSP119 were used to define cumulative exposure. For *P. vivax*, recent exposure was defined by responses to *Pv*RBP2b, *Pv*EBP, and *Pv*DBPRII, and historical exposure was determined by responses to *Pv*MSP119 and *Pv*AMA1 (Supplemental Table 1). The glutathione-S-transferase (GST) protein was included as a generic antigen to assess potential nonspecific binding to GST-tagged proteins. A 5-fold standard curve from a serum pool of hyper-immune individuals in Tanzania (CP3) was used to control for plate reproducibility. The *P. falciparum* WHO reference serum (NIBSC 10/198) was used as a positive control. Plasma samples from United Kingdom malaria-naive individuals donated by the former Public Health England were used as negative controls.

Plasma was diluted to a final concentration of 1:400 overnight at 4°C in Buffer B (1× phosphate-buffered saline [PBS], 0.05% Tween-20, 0.5% bovine serum albumin [BSA], 0.02% sodium azide, 0.1% casein, 0.5% polyvinyl alcohol, 0.5% poly-vinylpyrrolidone, and 15.25 *µ*g/mL *Escherichia coli* [*E. coli*] lysate to minimize nonspecific background against antigens expressed in *E. coli*). Immunoglobulin G (IgG) antibodies were quantified as described in Wu et al.^13^ Briefly, diluted plasma samples were incubated simultaneously with all coupled microspheres, washed, and then incubated with an R-phycoerythrin-conjugated goat anti-human IgG Fcγ-specific secondary antibody (Jackson ImmunoResearch [West Grove, PA, USA]; 109-116-098). After a further wash, a final 30-minute incubation was performed in Buffer A (1× PBS, 0.05% Tween-20, 0.5% BSA, 0.02% sodium azide). Plates were then washed once before being read on a MAGPIX^®^ bioanalyzer (Luminex Corp.). All incubations were performed for 90 minutes on a shaker in the dark unless otherwise stated. Washing steps were performed with 100 *µ*L per well in 1× PBS and 0.05% Tween, and both sample and secondary antibody additions were made at 50 *µ*L per well.

Quality control analyses were conducted in R Studio (R Foundation [Vienna, Austria]). Mean median fluorescence intensities (MFIs) from each positive control dilution point and three standard curve dilution points (high: 1:50, medium: 1:250, and low: 1:6250) were plotted using Levey–Jennings charts. Plates were repeated if more than two dilution points for more than two antigens fell outside the acceptable range of the mean ± 2 SDs. Background signals were examined for elevated noise, and GST mean MFIs were reviewed to monitor potential cross-reactivity.

### Data analyses.

All data analyses were performed using R (R Foundation).^14^ Antigen-specific IgG antibody responses were recorded as adjusted MFI. Dichotomous outcomes for seropositivity to each antigen were determined using unsupervised machine learning k-means clustering algorithms. The k-means algorithm was applied to the MFI values for each antigen independently, and k was set to two distinct clusters. The two clusters were assumed to represent a seronegative and a seropositive subpopulation, with the seronegative subpopulation assumed to be those with the lowest distribution of MFIs. A combined antibody response to recent or historical exposure to *P. falciparum or P. vivax* was subsequently calculated. If a participant tested positive for ≥1 historical *P. falciparum* antigens, they were classified as historically exposed (within the last 20 years). If a participant was seropositive for ≥2 recent *P. falciparum* antigens, they were classified as recently exposed (within the last 9 months). Similarly, if a participant tested positive for ≥1 historical *P. vivax* antigen, they were classified as historically exposed. If a participant was seropositive to ≥3 recent *P. vivax* antigens, they were classified as recently exposed (Supplemental Table 2).

Descriptive statistics were calculated using the demographic data collected in the survey. Historical data on malaria cases between 2006 and 2018 were extracted from Yapabandara et al.^6^ and plotted against *P. falciparum* historical exposure data.

## STATISTICAL ANALYSES

Age-specific seroprevalence data were plotted and fitted using a reverse catalytic model as previously described^15^^,^^16^ to estimate seroconversion rates (SCRs) for historical exposure to *P. falciparum* and *P. vivax*. Seroconversion rates provide an estimate of how many individuals seroconvert to become antibody-positive per year and serve as an alternative measure of the force of infection. Seroconversion rates were estimated using models with a single SCR and models that allowed for a change in the force of infection (two-step SCR) for an optimal fit to the observed prevalence. The time of change was selected by evaluating the two-step SCR models for each age and choosing the model that yielded the greatest improvement over the single-SCR model. If no changepoint improved the fit, the single-SCR model was selected. A log-likelihood ratio test indicative of the model’s fit to the data was used to compare the single- and two-step SCR models for each species.

Univariate binomial mixed-effects logistic regression models were run for each covariate for each exposure. Enumeration area was set as a random effect in all models to account for potential clustering of outcomes. The outcome of the models was an individual’s binary seropositive or seronegative status for historical or recent exposure to *P. falciparum* or *P. vivax*. A multivariate binomial mixed-effects logistic regression was developed for each exposure using forward selection. All models were adjusted a priori for known confounders (age and sex), with EA specified as a random effect. Covariates were added sequentially, and likelihood ratio tests were used to assess improvement in model fit compared with the preceding model. Covariates with likelihood ratio *P*-values <0.05 were retained in the final model.

### Spatial analysis.

The EA-level seroprevalence of historical and recent exposure to *P. falciparum* and *P. vivax* was calculated, and maps were created using QGIS (QGIS Association [Grüt, Switzerland]).^17^

Because the seroprevalence of recent exposure to *P. falciparum* and *P. vivax* was low, it was not suitable to explore their associations with current environmental conditions. Furthermore, temporal inconsistencies between historical exposure (within the last 20 years or more) and present-day environmental and climatic trends constrained the authors to a limited set of variables. Geospatial variables which are known to be associated with malaria transmission in Southeast Asia, were selected and temporally aligned with the study definition of historical exposure. For each EA, the mean elevation, which is temporally stable, and historical tree cover (percentage of canopy closure for vegetation over 5 m tall per 30-m grid cell) in 2000 were calculated. This is consistent with the study definition of historical exposure, Timor-Leste gaining independence, and the beginning of nationwide efforts to tackle malaria. Elevation and historical tree cover were extracted from open-source remote-sensing repositories.^18^^,^^19^

Linear regression models were used to analyze factors that may influence historical malaria exposure, with mean EA-level age-standardized seroprevalence of historical exposure to *P. falciparum* and *P. vivax* as outcomes. Age-standardized seroprevalence was used to account for potential differences in population age structure between EAs and to minimize bias in the spatial analysis. The overall study population was used as the standard to derive age-specific weights. Individuals were grouped into five age strata (0–9, 10–20, 21–32, 33–48, and >49 years), and age-specific seroprevalence within each EA was weighted by the corresponding proportion of individuals in the same strata in the study population. The weighted values were summed to estimate age-standardized EA-level seroprevalence.

Correlation matrices and Pearson’s correlation were used to assess relationships among EA-level seroprevalence quintiles for different exposure types. Analysis of the concordance between, for example, historical exposure to *P. falciparum* and *P. vivax* may help identify areas that were previously co-endemic for each species.

## RESULTS

### Survey.

A total of 4,631 people were recruited from all 13 municipalities in Timor-Leste (Figure 1). Dili, which contains the capital city, had the greatest number of participants (24.0%). There were more females (59.4%) than males (40.6%), and 81% of participants were over 22 years of age. History of travel abroad within 6 months or a lifetime, fever within 6 months, and lifetime history of malaria were all relatively low (1.1%, 1.2%, 9.0%, and 2.7%, respectively). A summary of the demographic characteristics of survey participants is provided in Table 1.

**Figure 1.**
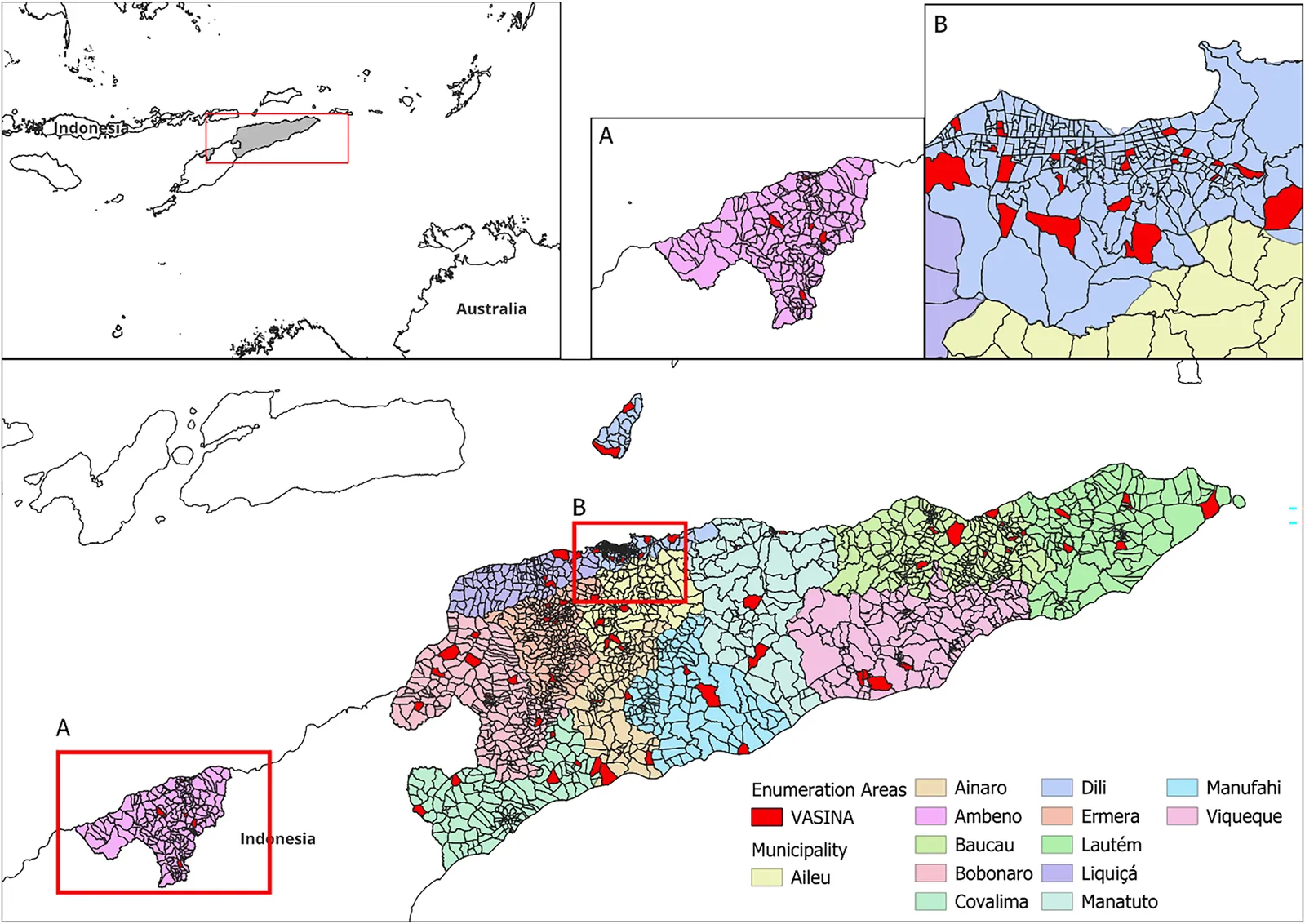
Map of Timor-Leste revealing the enumeration areas included in the Vaccine Preventable Disease Seroprevalence in a Nationwide Assessment of Timor-Leste survey within municipalities. (**A**) The exclave municipality of Ambeno and (**B**) the capital Dili are shown in the inset maps.

**Table 1:** Characteristics of Vaccine Preventable Disease Seroprevalence in a Nationwide Assessment of Timor-Leste survey participants

Variable	Proportion	Percentage (95% CI*)
Sex		
Female	2,753/4,631	59% (58%–61%)
Male	1,878/4,631	41% (39%–42%)
Age quintile (years)		
1–10	887/4,631	19% (18%–20%)
11–21	938/4,631	20% (19%–21%)
22–33	916/4,631	20% (19%–21%)
34–49	936/4,631	20% (19%–21%)
50–101	954/4,631	21% (19%–22%)
Setting		
Urban	3,436/4,627	74% (73%–75%)
Rural	1,195/4,627	26% (25%–27%)
Travel 6 months		
No	4575/4,631	99% (98%–99%)
Yes	52/4,631	1% (1%–1%)
Travel lifetime		
No	4,057/4,625	88% (87%–89%)
Yes	568/4,625	12% (11%–13%)
Fever 6 months		
No	4,201/4,619	91% (90%–92%)
Yes	418/4,619	9% (8%–10%)
Malaria history		
No	4,480/4,607	97% (97%–98%)
Yes	127/4,607	3% (2%–3%)
Municipality		
Aileu	180/4,631	4% (3%–4%)
Ainaro	282/4,631	6% (5%–7%)
Baucau	436/4,631	9% (9%–10%)
Bobonaro	378/4,631	8% (7%–9%)
Covalima	353/4,631	8% (7%–8%)
Dili	1,112/4,631	24% (23%–25%)
Ermera	327/4,631	7% (6%–8%)
Lautem	316/4,631	7% (6%–8%)
Liquica	214/4,631	5% (4%–5%)
Manatuto	246/4,631	5% (5%–6%)
Manufahi	176/4,631	4% (3%–4%)
Oecusse	295/4,631	6% (6%–7%)
Viqueque	316/4,631	7% (6%–8%)

Some variables do not add up to 4,631 because of missing data. Travel 6 months: participant had traveled outside of the country within the past 6 months. Travel lifetime: participant had traveled outside the country within their lifetime. Fever 6 months: participant had experienced fever within the past 6 months. Malaria: participant reported being infected with malaria in their lifetime.

* Percentages are shown with 95% CIs calculated using binomial approximations.

### Serology.

Seropositivity for recent exposure to *P. falciparum* and *P. vivax* was very low overall in the study population (1.4% [CI: 1%–2%] and 0.2% [CI: 0.1%–0.3%], respectively). Recent exposure was evenly distributed across all ages of the study population. Seropositivity to historical exposure for both species was higher (15.2% [CI: 14%–16%] and 5.1% [CI: 5%–6%], respectively; Table 2). There was a clear age pattern in historical seroprevalence for both species, with very low to no exposure in individuals born in the past 20 years (Figure 2) but evidence of exposure in those born before this time period, reflecting the country’s malaria endemicity in the past.^20^

**Table 2:** Individual-level risk factors for seropositivity to historical and recent exposure to *Plasmodium falciparum* and *Plasmodium vivax*

Variable	Level	*n*	*P. falciparum*						*P. vivax*			
			Historic			Recent			Historic			Recent
			Positive, *n* (%)	OR	*P*	Positive, *n* (%)	OR	*P*	Positive, *n* (%)	OR	*P*	Positive, *n* (%)
Overall	Overall	4,631	705 (15)	–	–	67 (1)	–	–	234 (5.1)	–	–	7 (0.2)
Age quintile (years)	1–10	4,631	9 (0)	Int	Int	8 (0)	Int	Int	17 (0.4)	Int	Int	0 (0)
	11–21	4,631	18 (0)	2.20	0.055	25 (1)	3.04	0.007	36 (0.8)	2.107	0.013	0 (0)
	22–33	4,631	83 (2)	10.67	0.000	11 (0)	1.38	0.499	30 (0.6)	1.754	0.071	1 (0)
	34–49	4,631	216 (5)	36.30	0.000	8 (0)	0.96	0.933	50 (1.1)	2.850	0.000	3 (0.1)
	50–101	4,631	379 (8)	90.11	0.000	15 (0)	1.90	0.152	101 (2.2)	6.146	0.000	3 (0.1)
Fever 6 months	No	4,619	651 (14)	Int	Int	60 (1)	Int	Int	216 (4.7)	Int	Int	7 (0.2)
	Yes	4,619	53 (1)	0.90	0.518	7 (0)	1.13	0.767	18 (0.4)	0.811	0.419	0 (0)
Malaria history	No	4,607	674 (15)	Int	Int	64 (1)	Int	Int	226 (4.9)	Int	Int	7 (0.2)
	Yes	4,607	30 (1)	2.29	0.000	3 (0)	1.72	0.375	6 (0.1)	1.037	0.933	0 (0)
Setting	Rural	4,631	615 (13)	Int	Int	36 (1)	Int	Int	179 (3.9)	Int	Int	7 (0.2)
	Urban	4,631	90 (2)	0.40	0.000	31 (1)	2.48	0.001	55 (1.2)	0.940	0.775	6 (0.1)
Sex	Female	4,631	377 (8)	Int	Int	44 (1)	Int	Int	140 (3.0)	Int	Int	1 (0)
	Male	4,631	328 (7)	1.37	0.000	23 (0)	0.78	0.353	94 (2.0)	0.988	0.932	2 (0)
Travel 6 months	No	4,627	689 (15)	Int	Int	65 (1)	Int	Int	228 (4.9)	Int	Int	5 (0.1)
	Yes	4,627	15 (0)	3.54	0.000	2 (0)	3.19	0.128	5 (0.1)	2.334	0.091	7 (0.2)
Travel lifetime	No	4,625	580 (13)	Int	Int	55 (1)	Int	Int	197 (4.3)	Int	Int	7 (0.2)
	Yes	4,625	124 (3)	2.52	0.000	12 (0)	1.64	0.147	37 (0.8)	1.535	0.036	0 (0)

Int = intercept; OR = odds ratio; *P. falciparum* = *Plasmodium falciparum*; *P. vivax* = *Plasmodium vivax*.

Results are from univariate models. A full table with 95% CIs and standard errors is provided in the Supplemental Information. Due to the very low number of seropositives to recent exposure to *P. vivax* (7/4,631), most univariate models did not converge and have not been included in this summary table.

**Figure 2.**
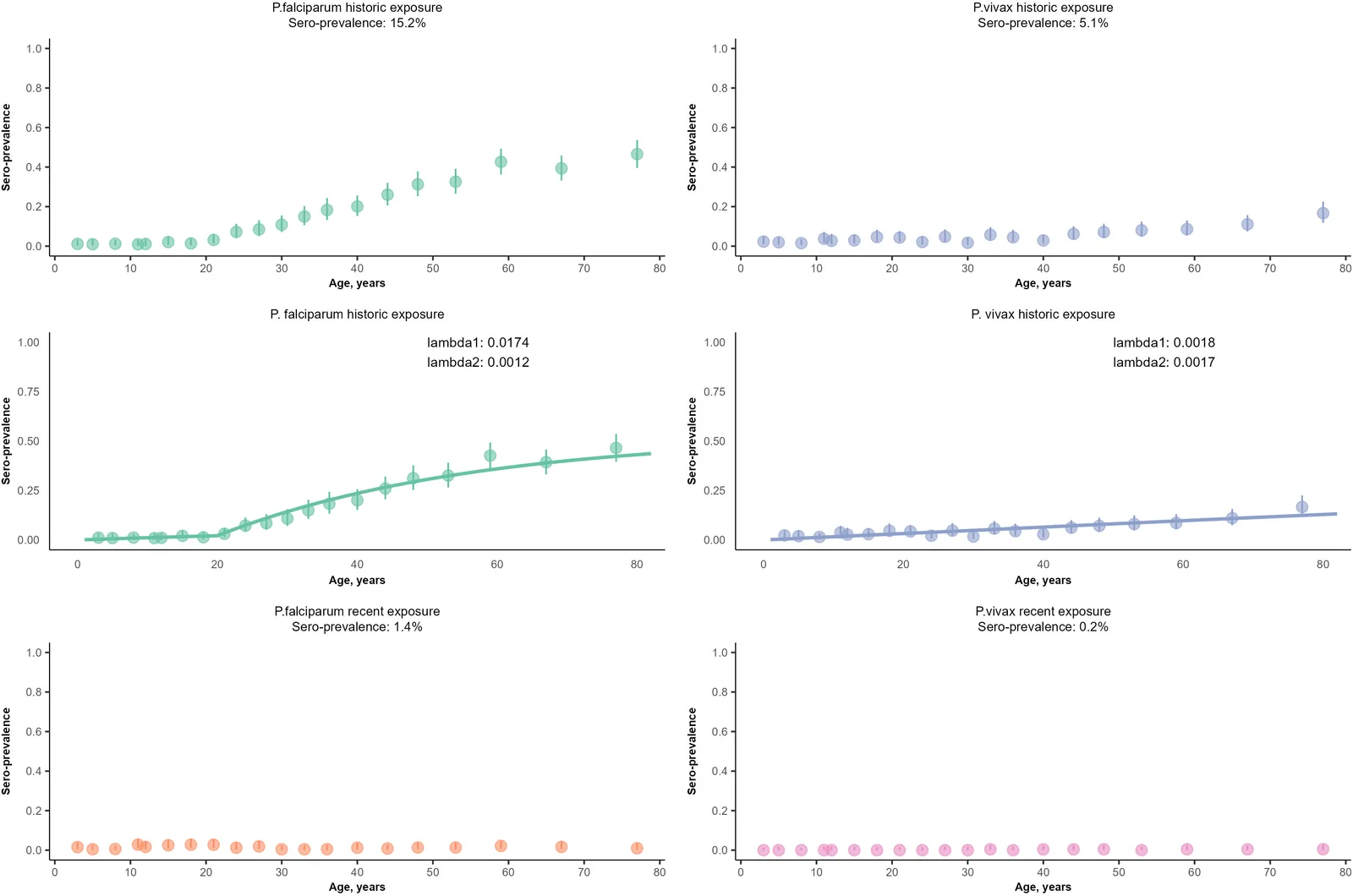
Age-stratified seroprevalence curves and seroconversion rate (SCR) curves. Top panel: Age-stratified seroprevalence curves for historical exposure to *Plasmodium falciparum* (*P. falciparum*) and *Plasmodium vivax* (*P. vivax*). Middle panel: SCR curves for historical exposure to *P. falciparum* and *P. vivax*. Bottom panel: Age-stratified seroprevalence curves for historical and recent exposure to *P. vivax*.

Two models to estimate the force of infection were compared: SCR1, with a single SCR, and SCR2, which allowed for a change in the force of infection at a specific time point. For *P. falciparum*, SCR2 was a better fit to the age-stratified seroprevalence data (SCR2 log-likelihood: −185.02; SCR1 log-likelihood: −300.58; *P* <0.05). SCR2 was fitted with a change in SCR at 20 years, with more than a 10-fold reduction in SCR between time points (λ = 0.017; λ = 0.001). For *P. vivax*, SCR1 and SCR2 yielded nearly identical log-likelihood values (−167.83 and −167.80, respectively), and a likelihood ratio test indicated no significant difference between the models (0.97), suggesting that both models fit the data similarly. There was a small change in the SCR across time points, and the SCR was low (λ = 0.0018 versus λ = 0.0017; Figure 2). The SCR for recent exposures was not calculated because of uniformly low levels of seroprevalence across all ages, which meant that the models did not converge to generate a credible fit.

Annual *P. falciparum* case data and milestones from the development of the NMCP from 2006 to 2018 were extracted from Yapabandara et al.^6^ These were plotted against the age-stratified *P. falciparum* seroprevalence in Figure 3.

**Figure 3.**
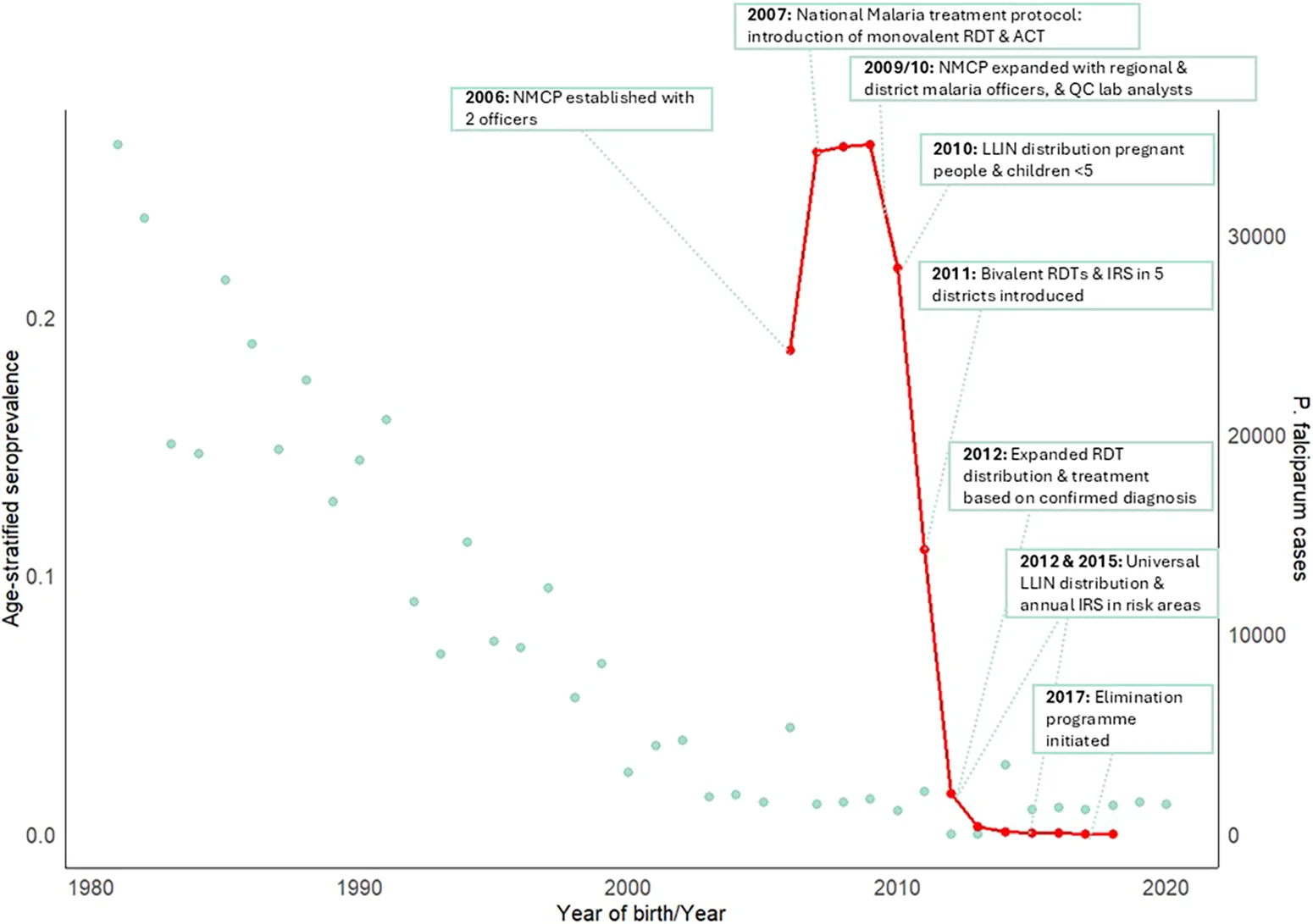
*Plasmodium falciparum* (*P. falciparum*) age-stratified seroprevalence (green dots) by year of birth and *P. falciparum* malaria case counts (red dotted line) by calendar year. The *x*-axis represents both the year of birth (for seroprevalence) and the year (for malaria case data), aligning age with temporal trends. Key malaria control and elimination interventions are annotated chronologically. The left *y*-axis represents seroprevalence, and the right *y*-axis represents annual *P. falciparum* case numbers. Adapted from Yapabandara et al.^6^

### Individual risk factors.

The results of the univariate logistic regression analysis are presented in Table 2. As expected, individuals in older age groups were more likely to be seropositive for antigens associated with historical exposure to both *P. falciparum* and *P. vivax* (Table 2). Additionally, for *P. falciparum*, an increased risk of historical exposure was observed in individuals who had a self-reported history of malaria (odds ratio [OR]: 2.3; 95% CI: 1.44–3.63; *P* <0.005), were male (OR: 1.37; 95% CI: 1.16–1.63; *P* <0.005), had traveled outside Timor-Leste in the past 6 months (OR: 3.54; 95% CI: 1.76–7.1; *P* <0.005), or had traveled outside Timore-Leste in their lifetime (OR: 2.52; 95% CI: 1.92–3.31; *P* <0.005). Individuals living in urban settings were significantly less at risk of historical exposure to *P. falciparum* (OR: 0.02; 95% CI: 0.26–0.6; *P* <0.005). For historical exposure to *P. vivax*, an increased risk of seroprevalence was observed in individuals who reported having traveled outside of Timor-Leste in their lifetime (OR: 1.53; 95% CI: 1.03–2.3; *P* = 0.04). For recent exposure to *P. falciparum*, individuals were significantly more at risk if they lived in an urban setting (OR: 2.48; 95% CI: 1.45–4.25; *P* <0.005) or if they were in the 11 to 21 years age quintile (OR: 3.04; 95% CI: 1.35–6.83; *P* = <0.005). Because of the low number of individuals who were seropositive for recent exposure to *P. vivax* (7/4,631), univariate models did not converge; therefore, the results are not reported in the table.

In the final multivariate model for historical exposure to *P. falciparum*, living in an urban setting was a protective factor (OR: 0.50; 95% CI: 0.31–0.81; *P* = 0.005), being male was a risk factor (OR: 1.6; 95% CI: 1.31–1.94; *P* <0.005), and older age quintiles were increasingly more likely to be seropositive (Figure 4). Similarly, for historical exposure to *P. vivax*, older age quintiles were most likely to be seropositive (Figure 4). For recent exposure to *P. falciparum*, risk was significantly higher in lower age quintiles, with multivariate models indicating the 11 to 21 years age group was at an increased risk (OR: 2.8; 95% CI: 1.24–6.29; *P* = 0.01), and living in an urban setting was associated with an increased risk (OR: 2.4; 95% CI: 1.4–4.2; *P* <0.005; Supplemental Table 3).

**Figure 4.**
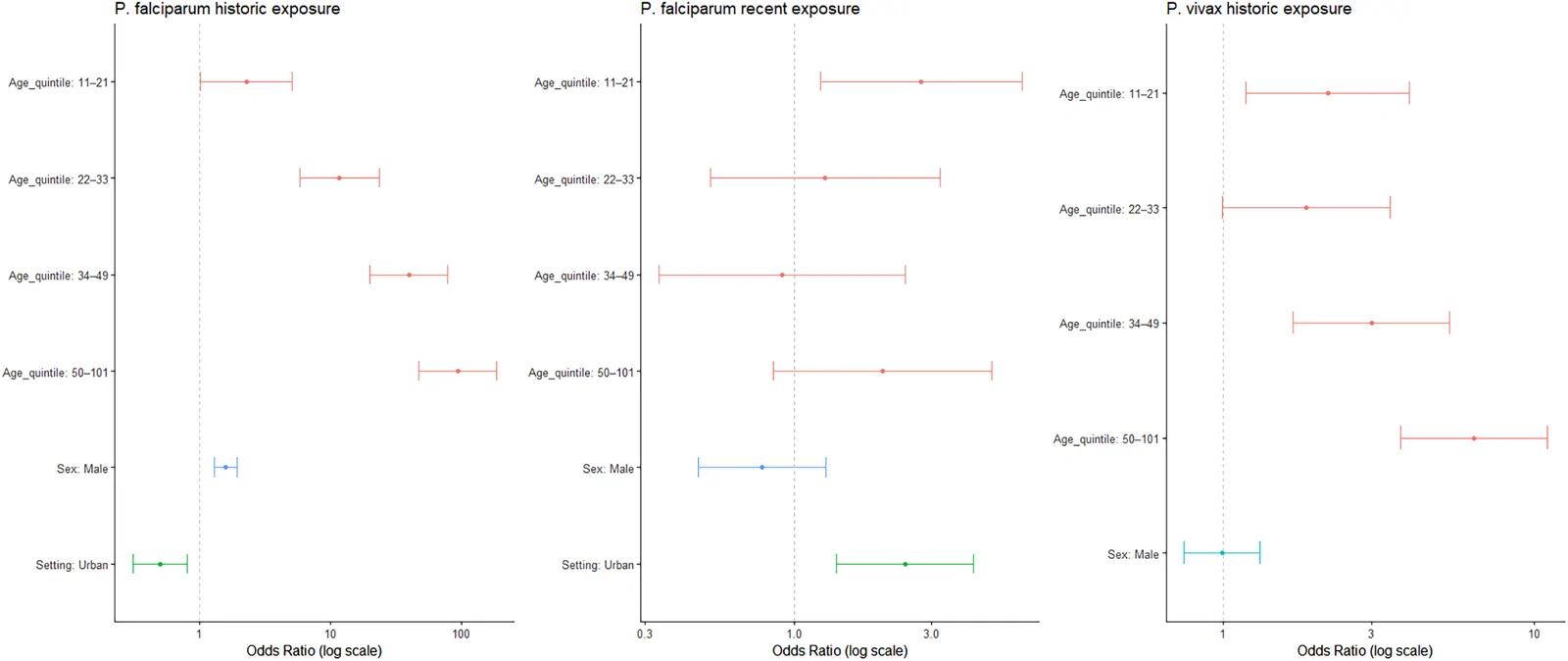
Forest plots of the final multivariate models for historical and recent exposure to *Plasmodium falciparum* and *Plasmodium vivax*. Variables with an adjusted odds ratio (OR) <1 are protective factors, and those with an OR >1 are risk factors. Variables with CIs spanning 1 are nonsignificant but were retained in the final model because they improved model performance (as indicated by Akaike’s information criterion [AIC]).

### Spatial analysis of EA level seroprevalence.

Countrywide seroprevalence is shown in Figure 5. The mean and range of EA-level seroprevalence of historical and recent exposure to *P. falciparum* and *P. vivax* in each municipality are presented in Table 3. Recent exposure to *P. falciparum* and *P. vivax* was uniformly low across the country, with the highest seroprevalence of recent *P. falciparum* exposure at 2.6% in the capital city of Dili. The highest seroprevalence of historical exposure to *P. falciparum* (31%) and *P. vivax* (10%) was observed in Viqueque on the southern coast of Timor-Leste, where the EAs are situated in areas of low elevation. For *P. falciparum*, Ermera (5%), and for *P. vivax*, Aileu and Manatuto (2%), all of which are located in upland areas in the center of the country, had the lowest historical exposure seroprevalence.

**Figure 5.**
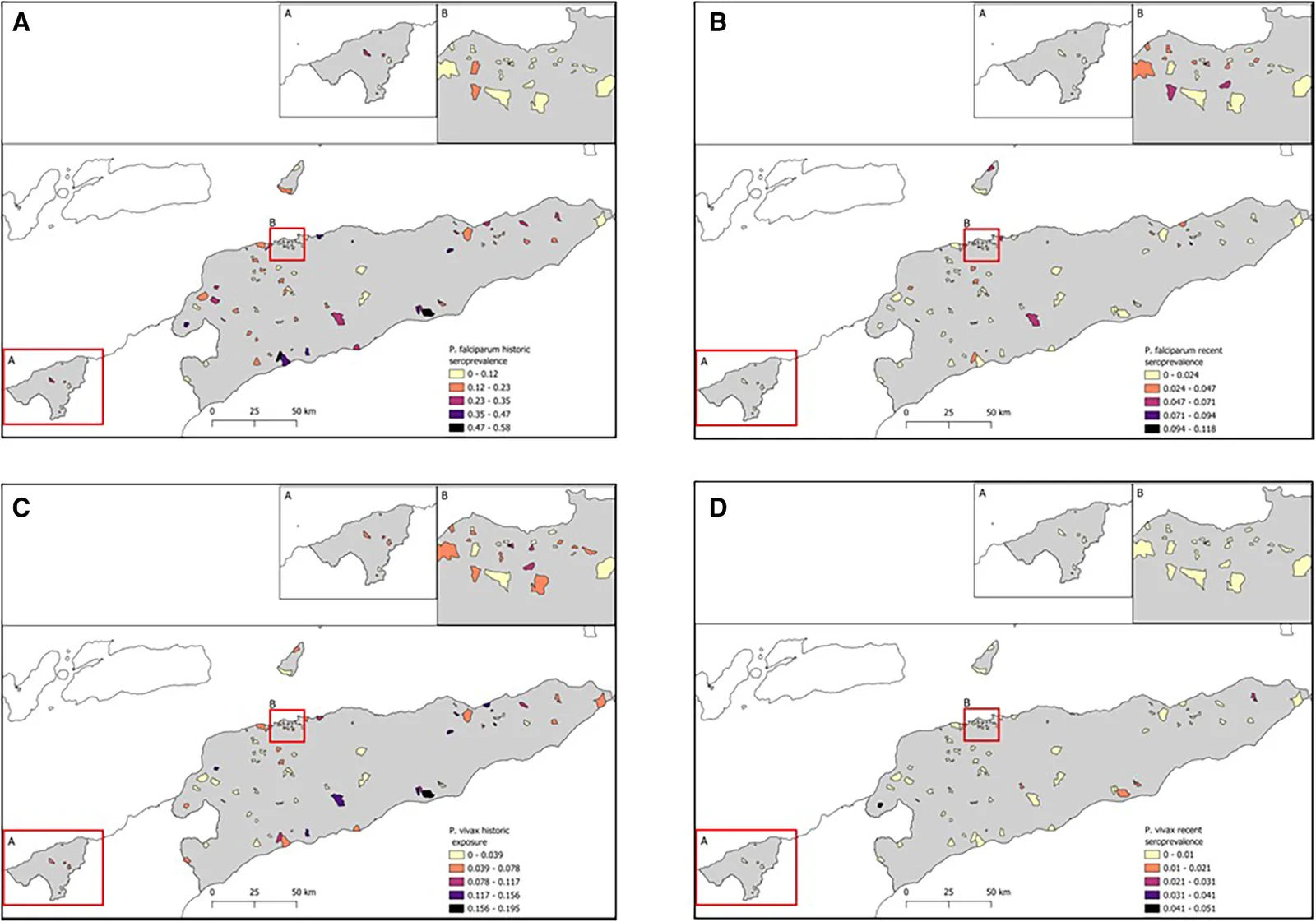
Maps of enumeration area-level seroprevalence of (**A**) historical exposure to *Plasmodium falciparum* (*P. falciparum*), (**B**) recent exposure to *P. falciparum*, (**C**) historical exposure to *Plasmodium vivax* (*P. vivax*), and (**D**) recent exposure to *P. vivax*. Note that scales are specific to each map.

**Table 3:** Enumeration area mean and range (minimum to maximum in brackets) seroprevalence for each municipality, including the number of enumeration areas surveyed per municipality

Municipality	EAs, *n*	Historical Exposure to *P. falciparum*	Recent Exposure to *P. falciparum*	Historical Exposure to *P. vivax*	Recent Exposure to *P. vivax*
Aileu	4	0.10 (0.02–0.18)	0.01 (0.00–0.03)	0.02 (0.00–0.05)	0.00 (0.00–0.00)
Ainaro	6	0.20 (0.03–0.44)	0.01 (0.00–0.03)	0.06 (0.00–0.16)	0.00 (0.00–0.00)
Baucau	11	0.18 (0.06–0.40)	0.02 (0.00–0.07)	0.09 (0.00–0.19)	0.00 (0.00–0.00)
Bobonaro	8	0.20 (0.07–0.36)	0.01 (0.00–0.04)	0.03 (0.00–0.12)	0.01 (0.00–0.05)
Covalima	6	0.18 (0.01–0.50)	0.01 (0.00–0.03)	0.03 (0.01–0.09)	0.00 (0.00–0.00)
Dili	30	0.08 (0.00–0.36)	0.03 (0.00–0.12)	0.05 (0.00–0.15)	0.00 (0.00–0.00)
Ermera	7	0.05 (0.00–0.14)	0.01 (0.00–0.05)	0.03 (0.00–0.06)	0.00 (0.00–0.00)
Lautém	5	0.20 (0.06–0.34)	0.01 (0.00–0.02)	0.05 (0.00–0.09)	0.00 (0.00–0.02)
Liquiçá	5	0.14 (0.05–0.26)	0.01 (0.00–0.05)	0.03 (0.00–0.05)	0.00 (0.00–0.00)
Manatuto	4	0.13 (0.07–0.24)	0.01 (0.00–0.02)	0.02 (0.00–0.03)	0.00 (0.00–0.01)
Manufahi	4	0.23 (0.16–0.29)	0.01 (0.00–0.06)	0.06 (0.03–0.12)	0.00 (0.00–0.02)
Oecusse	6	0.16 (0.04–0.32)	0.00 (0.00–0.02)	0.04 (0.00–0.07)	0.00 (0.00–0.00)
Viqueque	6	0.31 (0.12–0.58)	0.01 (0.00–0.02)	0.10 (0.02–0.19)	0.00 (0.00–0.02)

EA = enumeration area; *P. falciparum* = *Plasmodium falciparum*; *P. vivax* = *Plasmodium vivax*.

Enumeration area-level historical tree cover ranged from 0% to 88%, and mean elevation ranged from 8 m to 1,642 m (Supplemental Figure 2).

Age-weighted EA-level historical seroprevalence to *P. falciparum* decreased significantly with increasing elevation (β = −0.0001; 95% CI: −0.0001 to 0.0001; *P* <0.001), corresponding to a 0.01 decrease per 100 m increase in mean EA elevation. In contrast, seroprevalence increased with higher historical tree cover (β = 0.00195; 95% CI: 0.001–0.003; *P* <0.001), representing a 0.02 increase per 10% increase in average tree cover within an EA in 2000. The model explained 19% of the variation in EA-level *P. falciparum* seroprevalence (adjusted *R*^2^ = 0.19).

The same patterns were observed in the age-adjusted EA-level seroprevalence for *P. vivax*, with the model accounting for 11% of the variation in seroprevalence (adjusted *R*^2^ = 0.11). Historical seroprevalence of *P. vivax* decreased with increasing elevation, with a 0.05 reduction per 100 m increase in elevation (β = −0.0001; 95% CI: −0.0001 to −0.0000; *P* <0.001). Enumeration areas with higher historical tree cover had higher historical *P. vivax* seroprevalence, with a 0.06 increase in seroprevalence per 10% increase in tree cover (β = 0.001; 95% CI: 0.0001–0.001; *P* <0.05).

There were moderate positive correlations between historical exposure to *P. falciparum* and historical exposure to *P. vivax* (*r* = 0.56; *P* <0.001), as well as between historical exposure to *P. falciparum* and recent exposure to *P. vivax* (*r* = 0.52; *P* <0.001). A weak positive correlation was observed between historical exposure to *P. falciparum* and recent exposure to *P. vivax* (*r* = 0.24; *P* = 0.01; Supplemental Figure 1).

## DISCUSSION

In the present study, serological responses to malaria were analyzed as a proxy for exposure to infection in residents of Timor-Leste as an adjunct measure to support recent malaria elimination certification. The study involves the use of serology to characterize the historical and recent epidemiology of malaria in an elimination setting. Malaria was previously one of the top two priority diseases in the country,^5^ but transmission has dropped dramatically over the last 20 years through the efforts of the NMP. The epidemiological analysis of antibody responses to *P. falciparum* and *P. vivax* provides further strong evidence of the near-absence of recent exposure to infection across the population. Seropositivity to longer-lived antigens associated with historical exposure was higher among older populations, reflecting the higher levels of transmission when malaria was endemic. However, the seroprevalence of these antigens in those under 20 years of age and of antigens associated with recent exposure to infection was uniformly low.

The seroprevalence of antibodies to short-lived antigens associated with recent exposure to *P. falciparum* and *P. vivax* was low (1.4% and 0.2%, respectively) across all age groups, confirming the near-absence of exposure to these infections. This is consistent with the control measures and the recent certification of Timor-Leste as malaria-free.^9^ The low level of response to these antigens is also consistent with findings from other pre-elimination settings in the region. Using similar approaches of cross-sectional serological surveys, the seroprevalence of recent exposure to *P. falciparum* and *P. vivax* was 1% and 7%, respectively, in the Lao People’s Democratic Republic (PDR),^21^ and the seroprevalence of recent exposure to *P. falciparum* was 4% in Malaysian Borneo,^22^ with similar levels across all age groups. The average seroprevalence to antigens associated with historical exposure to *P. falciparum* and *P. vivax* in Timor-Leste was higher (15.2% and 5.1%, respectively), but these responses were almost exclusively observed in older individuals (i.e., those who were alive when malaria was endemic). Seroprevalence was close to 0% in the survey population under 20 years old. For *P. vivax*, this pattern was present but less pronounced, with a much lower overall seroprevalence. This finding is reflected in the historical data, with *P. vivax* making up ∼30% of malaria cases annually since 2006.^5^ Consistent with the study findings from Timor-Leste, in Malaysian Borneo, when the country was close to human malaria elimination in 2018, seroprevalence of historical exposure was estimated at 32% for *P. falciparum*, increasing with age,^22^ and 16% for *P. vivax*. In the Lao PDR, where *P. vivax* accounted for the majority of the malaria burden, the seroprevalence of historical exposure was 3% for *P. falciparum* and 22% for *P. vivax.* Here, exposure to *P. vivax* exhibited less pronounced age-related patterns, which is also similar to the study observations from Timor-Leste.^21^

The reverse catalytic model fitting results from the present study clearly reveal that the optimal fit for *P. falciparum* was a two-time step model with a change in SCR (a proxy for force of infection) at ∼20 years before the survey, which aligns with the notable dates of independence (2002) and the establishment of the NMP (2003). There was a greater than 10-fold difference in the force of infection pre- and post-interruption of transmission (λ = 0.017 and λ = 0.001, respectively). This estimate of the historical force of infection is likely an underestimate because antibody levels and seroprevalence will have waned in older individuals in the absence of exposure. Indeed, previous estimates of antibody half-lives, albeit based on ELISA methods, suggest half-lives of ∼20 years for the more immunogenic antigens associated with historical exposure.^23^ For *P. vivax*, the SCR model fit revealed a low and stable force of infection (λ = 0.002). This was to be expected because *P. vivax* has generally accounted for ∼30% of infections since records began in 2006.^6^ Together, the SCR estimates suggest low-level to absent malaria transmission, with 0.1% to 0.2% of the seronegative population becoming seropositive each year. Macalinao et al.^24^ used the same antigen panel and two-time step reverse catalytic models in an elimination setting in the Philippines and found results similar to those of the present study. In the province of Bataan, which had been declared malaria-free 5 years prior, the study revealed an estimated SCR between λ = 0.0019 and λ = 0.002 for historical exposure to *P. falciparum* and between λ = 0.0018 and λ = 0.0019 for historical exposure to *P. vivax* in age groups born after the interruption of transmission. These results confirmed the absence of recent transmission of *P. falciparum* and *P. vivax* in Bataan.

As described above, age was an individual-level factor associated with a higher seroprevalence against antigens associated with historical exposure to *P. falciparum* and *P. vivax.* Additionally, males were found to have higher odds of seropositivity. Although the sex-specific risk in Timor-Leste has not previously been reported to the best of the authors’ knowledge, de Almeida et al.^25^ reported the highest odds of infection in farmers compared with housewives, students, and other occupations in 2005. In Southeast Asian contexts, especially as countries near elimination, malaria (including zoonotic malaria) tends to be an occupational hazard in rural settings, specifically in working-age males involved in agriculture and forest work.^22^^,^^26^^–^^28^

Living in an urban setting was associated with a lower risk of historical exposure to *P. falciparum* and an increased risk of recent exposure, aligning with spatial patterns of seroprevalence. Historical exposure to *P. falciparum* was lowest in EAs in the capital city of Dili, likely reflecting the largely rural transmission. In contrast, the EAs in Dili, as well as those in Bacau, the second-largest urban capital, recorded the highest seroprevalences of recent exposure. Additionally, younger populations living in urban settings were found to be at higher risk of recent exposure to *P. falciparum*. This was unexpected, as malaria has not been described as an urban disease in Timor-Leste, and this younger population has had limited exposure because of a marked reduction in cases over the past 20 years. It is possible that increased human movement, including unreported travel or migration from the neighboring malaria-endemic West Timor in Indonesia, is associated with risk for importation of malaria cases.^5^ It is possible that migrants with malaria parasites or antibodies against malaria may move to urban areas for employment or study, causing this association. These findings may suggest a shifting risk profile as the country approaches elimination, in which surveillance among at-risk urban populations would be beneficial for preventing outbreaks or a resurgence of transmission in urban settings. It is important to note, however, that the CIs for these results from the multivariate models were wide, and the proportion of seropositives was very low (67/4,631), which limits the statistical precision and reliability of these findings.

There are limited data on the distribution of malaria in Timor-Leste.^5^ Historical records of malaria transmission reveal that transmission was absent or very low in areas over 500 m elevation, hypoendemic in the inland lowland areas, and mesoendemic on the coast.^5^^,^^29^^,^^30^ Elevation, distance from the coast, rice cultivation, and forest were included as factors in the country’s risk stratification implemented in 2007. Using the persistence of antibodies as markers of historical exposure, EA-level seroprevalence was estimated. The highest seroprevalence of historical exposure to both *P. falciparum* and *P. vivax* was recorded among EAs in areas of low elevation close to the coast in the Viqueque Municipality. Historical tree cover and elevation were associated with EA-level seroprevalence of historical exposure to *P. falciparum* and *P. vivax*. The risk of historical exposure to *P. falciparum* decreased with elevation, with an estimated 1% decrease in seroprevalence for every 100 m increase in average EA elevation. Additionally, higher levels of historical forest cover within an EA were associated with higher exposure, with a 0.2% increase in seroprevalence for every 10% increase in average tree cover within an EA in 2000. The positive correlation between historical exposure to *P. falciparum* and *P. vivax* suggests co-endemicity of these species during periods of higher transmission. As described above, the highest seroprevalence of recent exposure to *P. falciparum* was recorded in lowland coastal EAs in the municipalities that are home to Timor-Leste’s two major cities: Dili and Baucau. Although the present study involves classic measures such as elevation, historical forest cover, and distance from the coast, which may still be important predictors of malaria receptivity in Timor-Leste, other environmental and sociodemographic risk factors may increase in importance. It will be important to understand the risk factors associated with importation and any secondary cases to protect the most at-risk populations or areas and to prevent the resurgence of transmission.

There were several limitations to this study. Because of the large scale of the survey, the questionnaire for collecting sociodemographic details of participants was intentionally short. The survey was not originally designed as a malaria survey, and as a result, there were few malaria-specific questions, which limited the scope of the individual risk-factor assessment. Additionally, there were a more limited number of antigenic targets associated with recent exposure to *P. vivax* that have been recently described.^31^ This may have influenced the very low number of individuals seropositive to recent malaria exposure, which presented challenges for statistical modeling, particularly for *P. vivax*. Although this is a result of the encouraging progress in malaria control for Timor-Leste, it is an important limitation to acknowledge. Finally, although EA-level elevation and historical tree cover are factors known to impact malaria transmission in Southeast Asia, the study’s geospatial analyses were constrained by a limited set of covariates that could be aligned with the timing of historical exposure, potentially limiting the capture of other relevant environmental drivers.

## CONCLUSION

To conclude, the seroepidemiological results presented in the current study support the WHO’s certification of Timor-Leste as malaria-free by revealing the near-absence of ongoing malaria transmission. The combination of antigens associated with recent and long-term exposure provides important adjunct information on the current lack of exposure to malaria infection. Because official malaria records in the country only date back to 2006, malaria serology offers a unique advantage by providing insights into historical transmission patterns beyond the scope of traditional diagnostics. This enables some understanding of malaria dynamics in the country prior to independence and the establishment of the NMP. It also confirms the utility of serology in low-transmission and elimination malaria settings, especially when routine data are not available.

## Supplemental Materials

10.4269/ajtmh.25-0700 Supplemental Materials
