# Supplementary material for: Serology Confirms Near-Absence of Malaria Transmission in Timor-Leste
Source: Am J Trop Med Hyg. 2026 Aug 27;115(4):785–94. doi: 10.4269/ajtmh.25-0700 (PMC13550752; doi:10.4269/ajtmh.25-0700)
Supplement: Supplementary file 1 [file tpmd250700.SD1.pdf]

Supplementary Information

Supplementary Information Table 1: Antigen characteristics

Antigens	Abbreviation	Pathogens	Location	Exposure	Description	Reference
Early transcribed membrane protein 5 antigen 1 (N-terminal)	Etramp5Ag1	*P. falciparum*	Infected red blood cell (iRBC); Parasitophorous vacuole membrane	Recent	Marker of recent exposure based on blood stage infection; transmembrane protein exposed to immune system on iRBC surface and presumably following destruction; validated	30, 31
Gametocyte exported protein 18	Gexp18	*P. falciparum*	Gametocytes	Recent	Marker of recent exposure based on blood stage infection; transmembrane protein exposed to immune system on iRBC surface and presumably following destruction; validated	32
Heat shock protein 40, type II	HSP40	*P. falciparum*	iRBC;Gametocytes	Recent	Marker of recent exposure based on blood stage infection; transmembrane protein exposed to immune system on iRBC surface and gametocyte presumably following destruction; validated	32
Hypothetical protein 2	HYP2	*P. falciparum*	Trophozoites; Schizonts	Recent	Function unknown, validated	33

Apical membrane antigen 1	*Pf*AMA1	*P. falciparum*	Sporozoite/ merozoite	Historic	Marker of historical exposure based on blood stage infection; released from apical tip during RBC invasion. Essential to invasion process; validated	35
19 kDa fragment of merozoite surface protein 1 molecule	*Pf*MSP119	*P. falciparum*	Merozoite surface	Historic	Marker of historical exposure based on blood stage infection; Involved in invasion. Heterogeneous interaction with other merozoite proteins. Full function still to be determined. Validated.	36
Glutamate rich protein region 2	GLURP RII	*P. falciparum*	Merozoite	Historic	Marker of historical exposure based on blood stage; validated	34
19 kDa fragment of merozoite surface protein 1 molecule	Pv MSP 119	*P. vivax*	Merozoite surface	Recent	As for *P. falciparum*. Validated.	37
Reticulocyte-binding protein 2b	Pv RBP 2b	*P. vivax*	Merozoite	Historic	Reticulocyte binding protein homologe. Part of invasion machinery. Validated.	38

Supplementary Information Table 2: Supplementary Information Table 1: List of the name and number of antigens included in the combined antigen classifications for P. falciparum and P. vivax historic and recent exposure.

Species	Exposure	Antigens	Method of sero-classification	N antigens

P. falciparum	Historic	*Pf*MSP119<br>*Pf*AMA1<br>GLURPR2	K-means (k=2)	≥1
P. falciparum	Recent	Etramp5Ag1<br>GEXP18<br>HSP40<br>HYP2	K-means (k=2)	≥2
P. vivax	Historic	*Pv*MSP119<br>*Pv*AMA1	K-means (k=2)	≥1
P. vivax	Recent	*Pv*RBP2b<br>*Pv*EBP<br>*Pv*DBPII	K-means (k=2)	≥3

Supplementary Information Table 3: Multivariate model results

species	exposure	variable	level	OR	CI_lower	CI_upper	p	SE
P. falciparum	historic	Intercept	Intercept	0.005695	0.0027933	0.0116115	0.0000	0.00207
P. falciparum	historic	Age_quintile	11–21	2.278363	1.0171284	5.1035204	0.0454	0.937486
P. falciparum	historic	Age_quintile	22–33	11.76204	5.8357947	23.706401	0.0000	4.206012
P. falciparum	historic	Age_quintile	34–49	39.67978	20.02348	78.63193	0.0000	13.84639
P. falciparum	historic	Age_quintile	50–101	93.35509	47.225832	184.54249	0.0000	32.45908
P. falciparum	historic	Travel_lifetime	Yes	0.940135	0.6892136	1.2824085	0.6968	0.148924
P. falciparum	historic	Setting	Urban	0.505115	0.3123517	0.8168409	0.0054	0.123873
P. falciparum	historic	Sex	Male	1.591137	1.305408	1.939406	0.0000	0.160686
P. falciparum	historic	Travel_6_months	Yes	1.615068	0.7623604	3.4215369	0.2107	0.618609
P. falciparum	historic	Malaria_history	Yes	1.1365	0.6797281	1.9002197	0.6256	0.298056
P. falciparum	recent	Intercept	Intercept	0.005721	0.0025784	0.0126932	0.0000	0.002326
P. falciparum	recent	Setting	Urban	2.300611	1.3062008	4.0520669	0.0039	0.664434
P. falciparum	recent	Age_quintile	11–21	2.794282	1.2415152	6.2890976	0.0130	1.156572
P. falciparum	recent	Age_quintile	22–33	1.243868	0.4899457	3.1579186	0.6462	0.591284
P. falciparum	recent	Age_quintile	34–49	0.814943	0.2955424	2.2471655	0.6925	0.421744
P. falciparum	recent	Age_quintile	50–101	1.829978	0.745439	4.4924116	0.1872	0.838524

P. falciparum	recent	Travel_lifetime	Yes	1.691714	0.8433795	3.393369	0.1388	0.600812
P. vivax	historic	Intercept	Intercept	0.01621	0.0097161	0.0270447	0.0000	0.004233
P. vivax	historic	Age_quintile	11–21	2.096555	1.1615903	3.7840723	0.0140	0.631658
P. vivax	historic	Age_quintile	22–33	1.736765	0.9437742	3.196054	0.0761	0.540439
P. vivax	historic	Age_quintile	34–49	2.808561	1.5948908	4.9458018	0.0003	0.810868
P. vivax	historic	Age_quintile	50–101	5.999576	3.5213071	10.222032	0.0000	1.631108
P. vivax	historic	Travel_6_months	Yes	1.668252	0.6138617	4.5337014	0.3157	0.850962

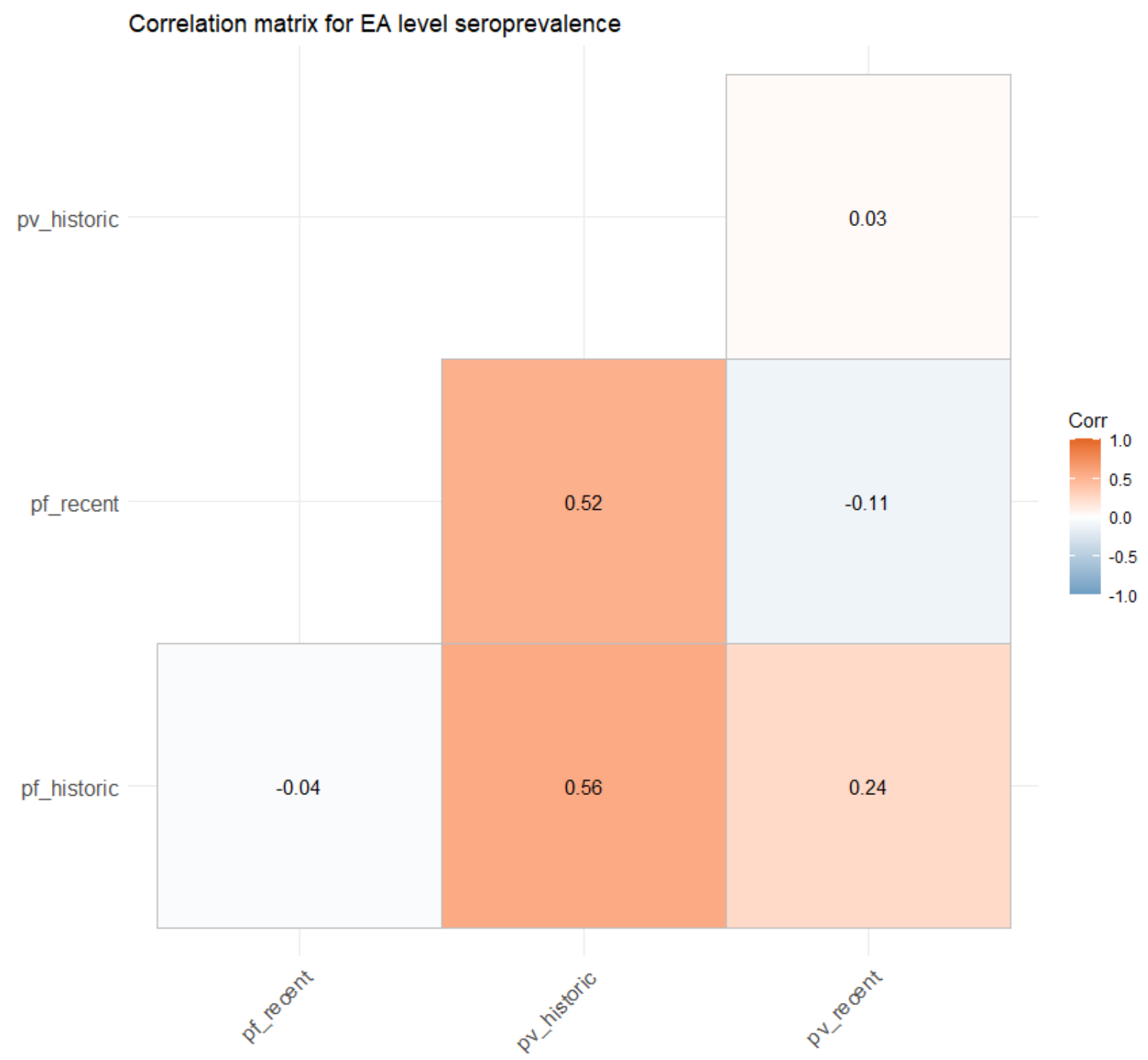

Supplementary Information Figure 1: Correlation matrix for EA-level seroprevalence. pf = P. falciparum, pv = P. vivax. The correlation coefficient r is in the center of the boxes. Positive correlations are red-toned, indicating that as one variable increases, the other also increases. . Negative correlations are blue-toned, indicating that as one variable decreases, the other also decreases. Darker shades indicate a stronger correlation (P value of Pearson's correlation coefficient)

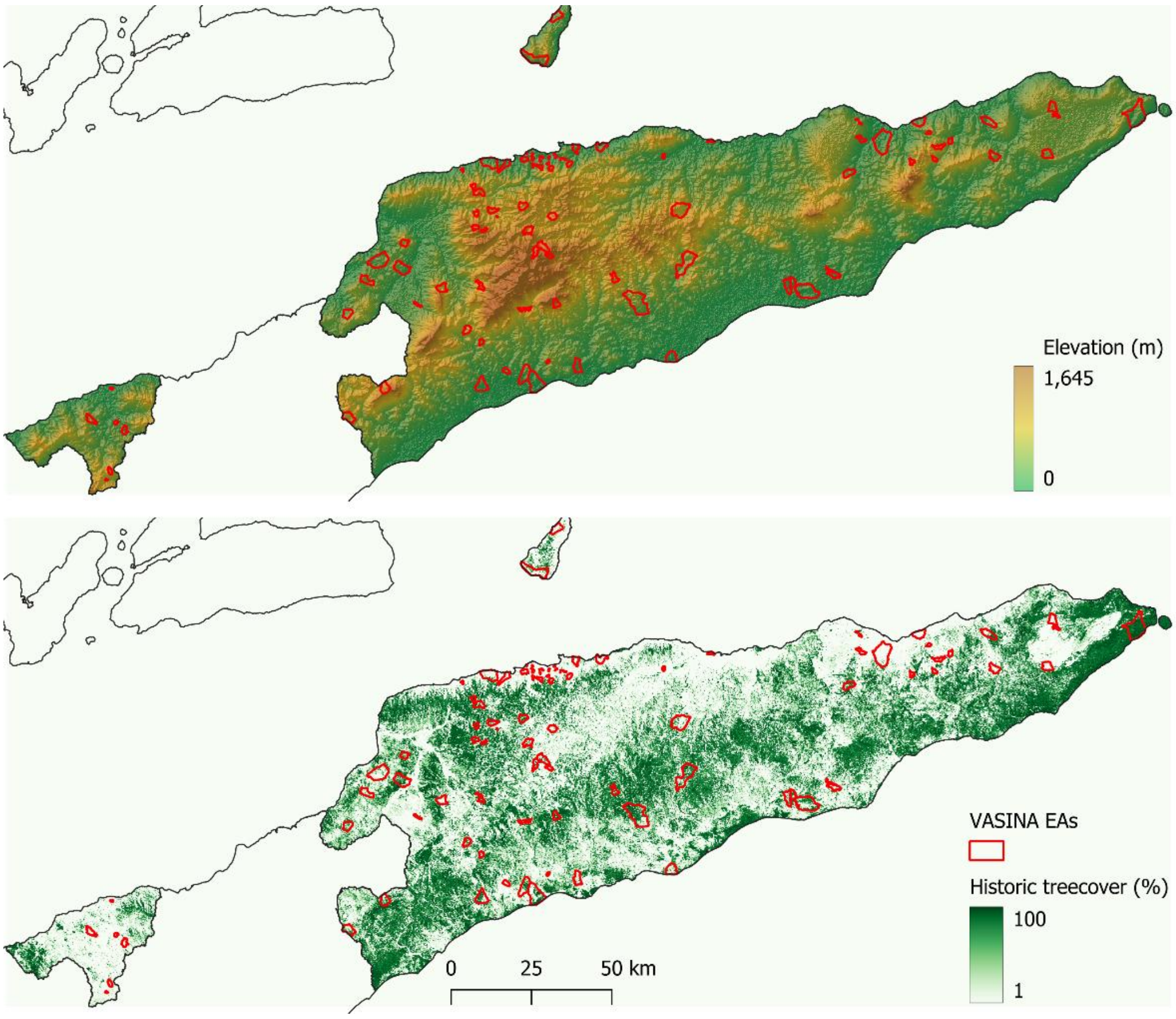

Supplementary Information Figure 2: Environmental covariates elevation (m) and historical treecover (% cover per pixel in 2000). Red boundaries denote the EAs included in the VASINA malaria serosurvey
